# FKSUDDAPre: A drug–disease association prediction framework based on F-TEST feature selection and AMDKSU resampling with interpretability analysis

**DOI:** 10.1371/journal.pcbi.1013947

**Published:** 2026-02-05

**Authors:** Yun Zuo, Chenyi Zhang, Ge Hua, Qiao Ning, Xiangrong Liu, Xiangxiang Zeng, Zhaohong Deng

**Affiliations:** 1 School of Artificial Intelligence and Computer Science, Jiangnan University and Engineering Research Center of Intelligent Technology for Healthcare, Ministry of Education, Wuxi, China; 2 Department of Computer Science and Technology, National Institute for Data Science in Health and Medicine, Xiamen Key Laboratory of Intelligent Storage and Computing, Xiamen University, Xiamen, China; 3 School of Information Science and Engineering, Hunan University, Yuelu District, Changsha, China; Jilin University, CHINA

## Abstract

In drug discovery and therapeutic research, the prediction of drug-disease associations (DDAs) holds significant scientific and clinical value. Drug molecules exert their effects by precisely identifying disease-related biological targets, systematically modulating the entire pharmacological process from absorption, distribution, and metabolism to final efficacy. Accurate prediction of drug-disease associations not only facilitates an in-depth understanding of molecular mechanisms of drug action but also provides critical theoretical foundations for drug repositioning and personalized medicine. While traditional prediction methods based on in vitro experiments and clinical statistics yield reliable results, they suffer from inherent drawbacks such as long development cycles, substantial resource consumption, and low throughput. In contrast, emerging machine learning techniques offer a promising solution to these bottlenecks, enabling the intelligent and efficient discovery of potential drug–disease association networks and significantly improving drug development efficiency. However, it is noteworthy that existing machine learning methods still face significant challenges in practical applications: the complexity of feature construction raises the threshold for data processing; data sparsity constrains the depth of information mining; and the pervasive issue of sample imbalance poses a severe challenge to the model’s predictive accuracy and generalization performance. In this study, we developed an efficient and accurate framework for drug-disease association prediction named FKSUDDAPre. The model employs a multi-modal feature fusion strategy: on one hand, it leverages an ensemble of Mol2vec and K- BERT to deeply capture the semantic features of drug molecular fingerprints; on the other hand, it integrates Medical Subject Headings (MeSH) with DeepWalk to effectively reduce the dimensionality of disease features while preserving their relational structure. To address the class imbalance problem, FKSUDDAPre designed an optimization algorithm called AMDKSU, which combined clustering with an improved distance metric strategy, significantly enhancing the discriminative power of the sample set. For data processing, F-test was employed for feature importance ranking, effectively reducing data dimensionality and improving model generalization. For the predictive architecture, FKSUDDAPre proposed a novel ensemble framework composed of XGBoost, Decision Tree, Random Forest, and HyperFast. By employing a dynamic weight allocation strategy, this ensemble effectively harnesses the complementary strengths of these models to achieve significantly enhanced predictive performance. Rigorous validation demonstrated the system’s outstanding performance across multiple evaluation metrics, with an average AUC of 0.9725, improving the AUC by approximately 3.88% compared to the best-performing baseline model. In the prediction of Alzheimer’s disease and Parkinson’s disease, 80% and 60% of the top 10 candidate drugs recommended by FKSUDDAPre, respectively, had been confirmed by literature, demonstrating the model’s good practical application potential. Furthermore, we conducted a LIME-based feature importance analysis on the model’s predictions, visualizing the correlations between features and the target variable to demonstrate the model’s interpretability. A cross-platform, user-friendly visualization tool had also been developed using the PyQt5 framework.

## Author summary

Drug repurposing offers a cost-effective alternative to traditional drug discovery, yet accurately predicting which existing drugs can treat specific diseases remains computationally challenging. In this study, we present FKSUDDAPre, a novel framework designed to identify potential drug-disease associations with high precision. Our approach is driven by three key innovations: first, the integration of pre-trained Large Language Models (specifically K-BERT) to capture deep semantic features of drug molecules; second, the development of the AMDKSU resampling algorithm, which effectively solves the critical issue of data imbalance to enhance model robustness; and third, the incorporation of HyperFast, a cutting-edge hypernetwork architecture, to boost classification performance. By combining these advanced components with a dynamic weighting strategy, FKSUDDAPre significantly outperforms existing baselines, achieving an average AUC of 0.9725. The framework’s practical utility was validated through case studies on Alzheimer’s and Parkinson’s diseases, where it successfully identified numerous literature-confirmed drug candidates. Furthermore, we prioritize transparency and usability by incorporating LIME-based interpretability analysis and providing a user-friendly visualization tool, making FKSUDDAPre a powerful resource for accelerating biomedical research.

## Introduction

Studies have shown that complex diseases—such as cancers and neurodegenerative disorders—often share common signaling pathways and molecular regulatory networks, laying a crucial theoretical foundation for the development of multi-target drugs and drug repurposing strategies [1–5]. Constructing high-precision drug–disease association maps not only accelerates the drug development process and reduces associated costs, but also significantly shortens the translational timeline from bench to bedside. Traditional validation methods that rely on in vitro experiments, animal models, and clinical trials are not only costly and time-consuming, but also suffer from limited coverage and scalability. Comparatively, AI-based computational prediction methods can systematically identify potential drug-disease associations by integrating multi-omics data. These approaches significantly reduce R&D costs while providing intelligent solutions for drug safety evaluation [6]. By constructing multi-scale feature spaces and fusing heterogeneous biomedical big data, advanced computational models can deeply analyze the mechanisms of drug action and their intrinsic connections to the onset and progression of diseases. This innovative research paradigm not only opens new avenues for breakthrough drug discovery but also provides powerful technological support for the clinical practice of precision medicine [7].

In recent years, numerous innovative computational methods have emerged in the field of drug-disease association prediction [8,9]. In 2011, Perlman et al. [10] pioneered the SITAR prediction framework, a model that ingeniously fused drug-drug [11] and gene-gene similarity information and introduced logistic regression for a weighted fusion of various similarity scores, laying a crucial foundation for target prediction research. In 2013, Wang et al. [12] proposed the forward-looking PreDR model, which established a generalized drug repositioning prediction framework by integrating multi-source heterogeneous data and utilizing support vector machines [13,14]. The following year, Oh et al. [15] took an alternative approach by developing an innovative random forest-based screening model derived from the topological features of the drug–disease network. As research progressed, in 2019, Yang et al. [16] introduced a boundary-blurring regularization technique and an overlapping matrix completion method, significantly enhancing model stability. In 2020, the same team [17] achieved another breakthrough with their MKDGRLS model, which innovatively introduced kernel methods and Laplacian regularization, substantially improving link prediction accuracy in bipartite networks. In the same year, Huang et al. [18] proposed the distinctive CMFMTL method, which combined collective matrix factorization with multi-task learning to reveal therapeutic mechanisms underlying drug–disease associations. In 2021, Jiang et al. [19] introduced the SAEROF, which integrated various similarity-based features and adopted a novel combination of sparse autoencoders and rotation forest, pushing association prediction performance to a new level.

Meanwhile, deep learning techniques have demonstrated groundbreaking progress in drug–disease association prediction [20–25]. In 2020, Xuan et al. [26] developed the GFPred prediction system by innovatively integrating graph convolutional autoencoders with attention mechanisms, significantly improving association recognition accuracy. In the following year, Wang et al. [27] designed the DCNN architecture, which combined convolutional attention mechanisms with a random forest classifier to construct an efficient prediction tool. In 2022, Zhao et al. [28] proposed the HINGRL model, which integrated drug, protein, and disease networks, and employed DeepWalk and autoencoders to learn graph structural features. In 2023, Gao et al. [29] introduced a co-contrastive learning strategy to build a GCL-based deep representation learning model. In 2024, Liu et al. [30] proposed the AMDGT, which jointly modeled multimodal information by combining multi-view similarity networks, Transformers, and multilayer perceptrons (MLPs). In the same year, He et al. [31] introduced the WMAGT, which enhanced graph neural network performance through the integration of graph attention mechanisms and neural collaborative filtering. Similarly, other studies have leveraged transformer-powered graph learning for tasks like identifying cancer genes across biological networks [32]. More recently, the field has begun to leverage Large Language Models (LLMs) to predict drug-drug interactions directly from biomedical knowledge graphs [33]. Indeed, the current research frontier is actively exploring structure-enhanced multimodal models to tackle complex cold-start scenarios and the challenge of deeper semantic understanding (SMPR, 2025) [34]. The continuous advancement of these cutting-edge techniques has not only revolutionized the paradigm of drug discovery but also provided robust methodological support for unraveling the mechanisms of complex diseases.

Despite significant progress, current drug-disease association prediction research faces three critical bottlenecks demanding urgent breakthroughs: Firstly, conventional feature engineering methodologies rely excessively on manually-designed representations, such as molecular fingerprints (e.g., ECFP), and on shallow semantic similarity metrics [35]. This approach is ill-equipped to accurately resolve the contextual semantics of molecular substructures and fails to adequately characterize the multi-level topological features of disease terminologies within the MeSH ontology tree. Consequently, this leads to a systematic loss of critical bio-semantic information. Secondly, data imbalance continues to constrain model performance. Current positive samples cover only a small fraction of the potential association space, resulting in frequent false negatives and misclassifications in prediction systems. Finally, the ‘curse of dimensionality’ associated with engineered features is particularly pronounced. The high-dimensional, sparse nature of molecular fingerprints, compounded by their inherent noise (such as hash collisions), not only significantly elevates computational complexity but also precipitates severe overfitting, especially when modelling with limited biomedical data. This ultimately compromises the model’s generalizability and, by extension, its clinical utility.

To address these challenges, we innovatively proposed the FKSUDDAPre framework, significantly enhancing drug-disease association prediction accuracy and robustness through integrated structural-semantic feature extraction, optimized sampling strategies, and multi-model ensemble learning. For drug feature extraction, FKSUDDAPre adopts an ensemble strategy combining Mol2vec and K-BERT to generate deep representations of molecular structures. This hybrid learning method effectively captures the semantic information of drug substructures and domain-specific knowledge embeddings. For disease feature extraction, FKSUDDAPre combines the structural information of the MeSH network with the DeepWalk algorithm to produce high-fidelity, low-dimensional embeddings of disease nodes, thereby preserving both the topological and semantic properties of diseases and comprehensively characterizing their ontological attributes and relationships. To tackle the critical issue of data imbalance, the study introduces an Adaptive Multi-Distance K-Means Similarity Undersampling algorithm (AMDKSU). This algorithm employs an intra-class neighbor retention strategy and multiple distance metrics to eliminate redundant and borderline samples, thus enhancing the balance between positive and negative samples in the dataset. Feature selection is performed using the F-test, which identifies low-dimensional features with strong discriminative power, effectively improving noise resistance and computational efficiency. For the predictive modelling stage, an ensemble of four heterogeneous models—XGBoost, Decision Tree, Random Forest, and HyperFast—is constructed. This architecture integrates the non-linear modelling capabilities of gradient boosting, the interpretability of decision trees, the inherent stability of bagging, and the rapid, meta-learning-based inference of HyperFast, further enhancing performance on the complex task of predicting DDA relationships. Experimental validation via 10-fold cross-validation confirms significant superiority across all metrics, establishing FKSUDDAPre as a reliable computational tool for drug repositioning.

**Fig 1:**
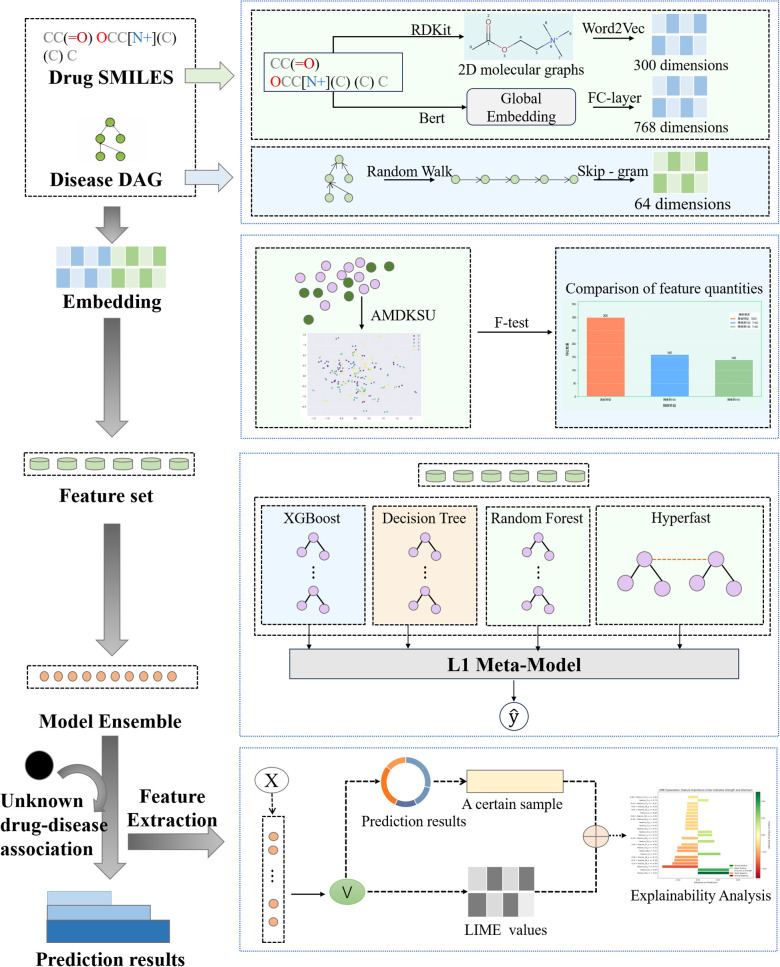
Framework of FKSUDDAPre.

As shown in Fig 1, the FKSUDDAPre framework comprises four core modules: feature extraction, balanced dataset construction, key feature selection, and ensemble learning prediction. The model takes as input the SMILES representations of drugs and a disease network structure constructed from MeSH terms, represented as a directed acyclic graph (DAG). The output is the predicted probability or binary classification result of a drug–disease association. Firstly, in the feature extraction module, Mol2vec and K-BERT are jointly applied to the SMILES sequences to perform comprehensive representation learning, producing enriched molecular embeddings that effectively capture the chemical semantics, domain knowledge, and contextual substructure information of drug molecules. For disease data, the DeepWalk algorithm is used to perform multi-scale random walks on the MeSH relational graph, generating 64-dimensional topological embeddings that fully preserve the hierarchical structure of disease nodes within the medical ontology. Subsequently, in the balanced dataset construction module, the concatenated feature vectors of initial drug–disease pairs are processed using an improved KSU (K-means Similarity Undersampling) strategy. This module conducts K-means clustering to analyze sample distribution and dynamically selects the distance metric that best fits the structure of the feature space. From each cluster, the most representative centroid samples are retained, effectively reducing redundancy and noise while mitigating the issue of severe class imbalance. Then, the key feature selection module applies the F-test to evaluate the discriminative power of all concatenated features by computing the variance ratio between positive and negative samples. The most informative features are selected for model training. Finally, the prediction system integrates four base learners: XGBoost, Decision Tree, Random Forest, and HyperFast. For the model validation stage, a top-k retrieval scheme is specifically designed, involving dual verification against authoritative databases such as the CTD and DrugBank to ensure the biomedical reliability of the predicted associations. In addition, we provide a user-friendly visualization tool to facilitate result interpretation and practical application, with example interface screenshots shown in the Appendix.

## Materials and methods

### Benchmark dataset

Comparative Toxicgenomics Database (CTD) [36] is an authoritative public bioinformatics platform that plays a crucial role in uncovering the potential mechanisms linking environmental chemicals to human diseases. By systematically integrating experimentally validated chemical–disease associations and leveraging bioinformatics prediction methods, CTD provides valuable support for environmental health research. Notably, its collection of literature-confirmed drug–disease interaction data is especially valuable for association prediction studies. DrugBank [37], a globally recognized drug knowledgebase, not only offers comprehensive records of molecular properties but also provides a detailed network of drug actions, including drug targets, metabolic enzymes, and drug–drug interactions. It serves as an essential reference for drug development and clinical pharmacology. MeSH (Medical Subject Heading) [38] thesaurus system, developed by the U.S. National Library of Medicine (NLM), employs a strictly standardized hierarchical classification system. It provides professional terminology standards for indexing and retrieving biomedical literature, significantly enhancing the accuracy and efficiency of medical information retrieval.

The dataset used in this study was derived from the work of Zhang et al. [39]. To mitigate the impact of data sparsity on prediction outcomes, we selected drug and disease entries from the CTD database that each had more than 10 associated relationships. After this rigorous screening, the final dataset was constructed, containing 269 drugs, 598 diseases, and their 18,416 verified positive associations. Additionally, the following key data sources were systematically integrated: 1) SMILES molecular fingerprint data for 269 drugs, extracted from the DrugBank database. 2) Hierarchical MeSH tree codes for 598 diseases, used for computing semantic similarities between disease terms.

Following the principle of theoretical association space integrity, we explicitly define the full combinatorial space of potential drug–disease pairs as 160,862. Among these, 18,416 represent confirmed positive associations, accounting for 11.4% of the entire space. The remaining 142,446 unverified pairs form the original negative sample pool. Recognizing the possibility that some unverified pairs may represent undiscovered true associations, we avoid naively treating them as true negatives. To ensure both class balance and biological plausibility, we adopt the KSU algorithm to select 18,416 unverified drug–disease pairs from the negative sample pool. This results in a balanced training set with a 1:1 ratio of positive and negative examples. This strategy not only maintains the theoretical space’s integrity and alleviates class imbalance, but also avoids the oversimplified assumption of non-association for unverified pairs—thus providing a scientifically sound foundation for model training.

### Feature extraction methods

In drug–disease association prediction tasks, feature extraction plays a pivotal role in model construction, aiming to transform heterogeneous biomedical entities into low-dimensional, dense, and computationally tractable vector representations [40]. Traditional methods, which rely on hand-crafted descriptors (such as molecular fingerprints or disease codes), suffer from limitations like being high-dimensional and sparse, a lack of semantic information, and poor transferability. With the advancement of deep learning, distributed representation learning has emerged as a mainstream paradigm. Through large-scale unsupervised pretraining, this approach effectively captures the underlying structural relationships and semantic similarities between biomedical entities. Such methods adhere to two fundamental principles: they must fully preserve the topological relationships of the original data (e.g., the substructural connectivity of drug molecules and the hierarchy of the disease MeSH tree), and they must ensure that semantically similar entities are placed in close proximity in the vector space (i.e., they receive adjacent embeddings). In this context, MeSHHeading2vec serves as a typical representative of this paradigm for disease representation. For drug representation, a hybrid strategy integrating Mol2vec and K-BERT is adopted. This strategy fuses information derived from chemical graph structures (via Mol2vec) and knowledge-based SMILES sequences (via K-BERT), aiming to construct a comprehensive and generalized feature representation capable of robustly supporting downstream prediction tasks.

### Drug feature extraction based on Mol2vec and K-BERT

This study leverages the complementary feature learning frameworks of Mol2vec and K-BERT to achieve a unified representation of drug molecules, capturing both local structural details and global semantic information.

We employ Mol2vec [41] to extract feature representations from drug SMILES sequences. This technique transforms molecular structures into chemically meaningful feature vectors through an innovative molecular vectorization pipeline. Inspired by the Word2Vec framework in natural language processing, Mol2vec deconstructs molecular topology and analyzes local atomic environments to construct a chemically relevant “molecular vocabulary.” It then applies the Skip-gram algorithm to learn contextual relationships between substructures, enabling efficient encoding of molecular features. The implementation process consists of three key steps:

1. Molecular structure parsing and substructure generation: The Morgan fingerprint algorithm is used to extract local structural features centered on each atom within a defined radius (radius = 1). Each atomic environment is represented by a unique substructure identifier (e.g., “C-1” denotes a carbon atom in a specific local context). The entire molecule is thus converted into a sequence of substructures, such as [‘C-1’,’O-3’,’N-5’,...].
2. Substructure vector space modeling: Based on a large-scale molecular corpus, a Word2Vec model is trained on a large-scale molecular dataset using the Skip-gram algorithm (with a context window of 10). It learns vector representations by modeling the contextual relationships between substructures, resulting in a fixed-length vector for each unique substructure.
3. Molecular vector aggregation: For each molecule, a molecule-level vector is generated by calculating a weighted average of the vectors for all of its constituent substructures. This process results in a final 300-dimensional global feature vector that represents the drug’s overall chemical characteristics. This feature vector implicitly encodes information such as functional groups and local chemical environments through the contextual relationships of its substructures. This approach avoids the need for hand-crafted descriptors (like LogP or molecular weight). Moreover, the pre-trained model offers high transferability to tasks with smaller datasets and allows for the analysis of molecular features through their constituent substructure vectors. These advantages significantly enhance both the representational power for drug structures and the effectiveness of predictive models.

Concurrently, the knowledge-based BERT model, K-BERT [42], is introduced to enhance global molecular semantic representation. Diverging from the initial multi-task design, during the actual pre-training phase, the model takes tokenized SMILES embeddings as input. It utilizes a Transformer encoder to learn sequence representations and employs a fully connected layer to predict predefined molecular fingerprints. This mechanism enables the model to directly learn the mapping between structural patterns and holistic chemical semantics. Pre-trained on approximately 1.8 million molecules from the ChEMBL database, K-BERT employs a six-layer Transformer architecture (hidden dimension = 768, 12 attention heads). The resulting embeddings (K-BERT-FP) effectively complement traditional fingerprints by capturing high-order characteristics—such as molecular size and chirality—that are often difficult for conventional descriptors to cover. Finally, the local substructure embeddings from Mol2vec are concatenated with the global molecular embeddings from K-BERT to form the definitive drug feature vector. This fused representation not only preserves fine-grained topological information but also encapsulates global semantics and fingerprint-level chemical features, providing a comprehensive, transferable, and interpretable input representation for downstream drug–disease association prediction.

### Disease feature extraction based on MeSHHeading2vec

DeepWalk [43] graph embedding algorithm is employed to extract features from the Directed Acyclic Graph (DAG) structure of diseases, in order to fully capture their hierarchical dependencies and global semantic structure within the Medical Subject Headings (MeSH) system. Specifically, for each disease node D, based on its MeSH TreeNumber code, a hierarchical backtracking strategy is applied: by progressively truncating the tail segments of the TreeNumber, a DAG structure containing all ancestor nodes of disease D is systematically constructed. This hierarchy-based decomposition approach not only accurately depicts the inheritance relationships among disease concepts but also effectively captures potential semantic association networks. At the algorithmic level, DeepWalk uses a carefully designed random walk strategy combined with neural network modeling to transform complex graph structural information into distributed representations in a low-dimensional vector space. The process consists of two main stages:

(1) Random Walk Generator: Define the disease graph as G= (V, E), where V is the set of all disease nodes, and E represents the edges between nodes. Starting from each disease node $V_{\text{0}}$, DeepWalk performs several truncated random walks to generate node sequences of length T:

$$\begin{array}{c} {\text{w}}_{\text{i}}=\left({\text{v}}_{\text{0},}{\text{v}}_{\text{1},}{\text{v}}_{\text{2}}\cdots {\text{v}}_{\text{T}-\text{1}}\right) \end{array}$$ (1)

Where Vrt+1+1 is uniformly sampled from the set of neighbors N~($V_{t}$) of node $V_{t}$, with the transition probability defined as:



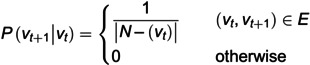

(2)

(2) Node Embedding Learning: For each random walk sequence w, the Skip-Gram model is used to optimize the following objective function:

max∑t=0T−1∑rj=−cj≠0clog P(vt+j|vt) (3)

This process maps each disease node into a dense vector of dimension d = 64, such that topologically similar nodes (e.g., direct parent-child diseases or those sharing common ancestors) are distributed closely in the embedding space. The model learns low-dimensional representations for each disease node by predicting the context neighbors of the current node. As a result, structurally similar or hierarchically related disease nodes are mapped to nearby regions in the vector space. In a DAG structure, diseases are not only connected via direct parent-child relationships but may also exhibit complex higher-order semantic dependencies through multiple paths. DeepWalk preserves both local adjacency information and global topological structure during the embedding process, making it particularly well-suited for knowledge graphs like MeSH, which contain strong hierarchical semantics. Ultimately, the model encodes each disease node into a 64-dimensional embedding vector, which serves as the disease semantic feature input for the downstream drug-disease association prediction task.

### K-means similarity undersampling algorithm based on adaptive multi-distance metric mechanism

In datasets for drug-disease association analysis, a significant class imbalance problem exists between positive and negative samples, with the ratio of associated to unassociated pairs reaching as high as 1:7. This imbalance in data distribution can lead traditional classification algorithms to become overly biased toward the majority class, thereby neglecting minority class samples that may possess critical research value. To address this critical challenge, this study innovatively proposed an improved KSU algorithm framework: AMDKSU (AMDKSU, K-means similarity undersampling algorithm based on adaptive multi-distance metric mechanism). By integrating an adaptive multi-distance metric mechanism with a dynamic group sampling technique, this algorithm effectively enhances the robustness of sample balancing and strengthens the discriminative power of the training data’s features. This lays a solid foundation for the subsequent construction of a high-precision drug-disease association prediction model.

KSU undersampling is an efficient sample selection technique whose core idea is to filter out the most representative samples using distance metrics. The method first employs K-means clustering to partition the majority class samples into k clusters. Subsequently, it rearranges sample pairs within each cluster based on their distance and dissimilarity matrices. During the implementation, the system identifies the N pairs of samples with the highest similarity and randomly removes one sample from each pair. This innovative process not only effectively preserves the distributional characteristics of the majority class but also significantly balances the sample size disparity between classes, thereby enhancing the classification model’s performance. The pseudocode of the algorithm is presented as follows.

**Algorithm 1. KSU Undersampling Algorithm.**

***Input:***

X: Feature matrix, where each row represents a sample

y: Label vector corresponding to each sample

***Output:***

X_resampled: Undersampled feature matrix

y_resampled: Undersampled label vector

***1. Initialize edited_samples as an empty list***

***2. FOR EACH*** *sample i* ***in*** *X* ***DO***

***3.***    Compute the Euclidean distance between sample i and all other samples j ≠ I: dis(i,j) = √(Σ(x_i- x_j)^2)

***4.***    Find the indices of the k nearest neighbors of sample i: neighbors_index

***5.***    Count the number of samples in neighbors_index that belong to a different class: count_diff_class

***6.    IF*** count_diff_class >= k/ 2 ***THEN***

***7.***        Add the index of sample *i* to edited_samples

***8.    END IF***

***9.*** Select samples based on the edited_samples list to create the undersampled feature matrix X_resampled and label vector y_resampled

***10. RETURN*** X_resampled, y_resampled

To better select representative samples, we introduces a grouped sampling strategy. Specifically, majority class samples are grouped based on drug molecular structures, and KSU undersampling is independently applied within each group to ensure local balance, thereby achieving global control over sample size. Concurrently, the approach adaptively utilizes five distinct distance metrics: Euclidean, Hamming, Chebyshev, Manhattan, and Minkowski. Their definitions and formulas are presented in Equations (4)–(8), respectively:


$$d_{E}(x,y)=\sqrt{\sum_{i=\text{1}}^{n}{\left(\chi_{\dot{l}}-y_{i}\right)}^{\text{2}}}$$ (4)

Euclidean Distance is Suitable for numerical, continuous feature spaces. it measures the straight-line distance between two points.


$$d_{H}(x,y)=\sum_{i=\text{1}}^{n}\;𝐈\left(x_{i}\neq y_{i}\right)$$ (5)

Hamming Distance is applicable to discrete or binary feature spaces. it counts the number of positions at which the corresponding elements of two vectors are different. I (⋅) is the indicator function, which equals 1 if the condition is true, and 0 otherwise.


$$d_{C}(x,y)=max\left|x_{i}-y_{i}\right|$$ (6)

Chebyshev Distance measures the maximum difference between two vectors along any coordinate dimension, making it suitable for scenarios where extreme values are significant.


$$d_{M}(x,y)=\sum_{i=\text{1}}^{n}\left|x_{i}-y_{i}\right|\;$$ (7)

Manhattan Distance is also known as the city block distance or L1 distance. It measures the sum of the absolute differences of the coordinates of two points.


$$d_{p}(x,y)={\left(\sum_{i=\text{1}}^{n}{\left|x_{i}-y_{i}\right|}^{p}\right)}^{\frac{\text{1}}{p}}$$ (8)

Minkowski Distance is generalized form of distance metrics. The parameter p controls the calculation, allowing it to represent several classic distances.

### Key feature extraction based on F-TEST

**Fig 2:**
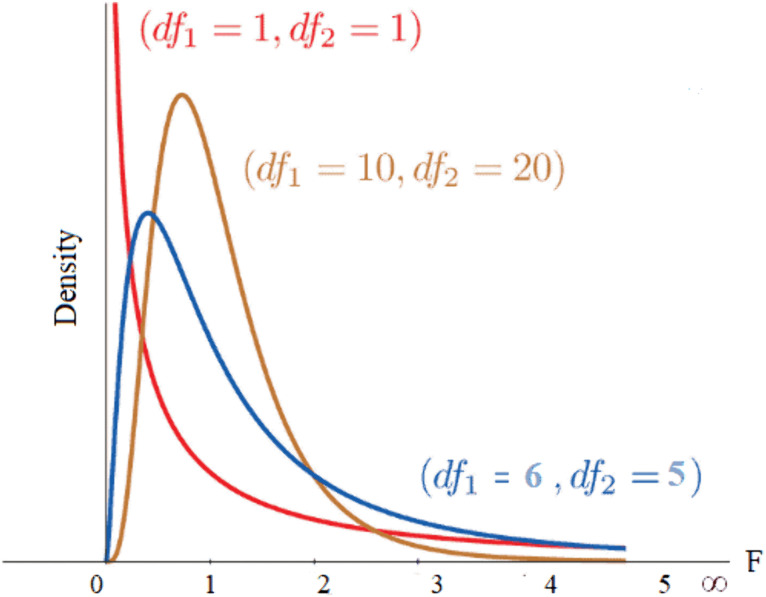
Density function of the F-distribution.

The F-test was selected as the feature selection method in this study because it provides a simple yet powerful statistical criterion for measuring the linear dependency between continuous features and categorical outcomes. Compared with more complex wrapper or embedded methods, the F-test offers high computational efficiency and strong robustness, making it particularly suitable for high-dimensional biological data. Moreover, as a classical and interpretable statistical approach, it enables transparent evaluation of feature importance, aligning with our goal of building an efficient and explainable prediction framework. F-test is employed to perform feature selection on drug features. As a filter method based on statistical inference, the F-test is essentially an analysis of variance (ANOVA) technique used to evaluate the strength of linear correlation between individual continuous features and categorical variables. As illustrated in Fig 2, the F-distribution shows the probability density functions corresponding to different F-values.

During the feature selection process, the F-test compares the variance of different classes across each feature dimension to assess the relationship between each feature and the target variable (e.g., drug–disease association). Specifically, the F-value is defined as the ratio of between-group variance to within-group variance, reflecting whether the feature exhibits statistically significant differences across categories. The basic formula is given in Equation (9).


$$\begin{array}{c} \text{F}=\frac{\text{Between}-\text{group}\;\text{variance}}{\text{Within}-\text{group}\;\text{variance}}=\;\frac{\frac{\sum_{\text{i}=\text{1}}^{\text{k}}{\text{n}}_{\text{i}{\left(\overset{―}{{\text{X}}_{\text{i}}}-\overset{―}{\text{X}}\right)}^{\text{2}}}}{\text{k}-\text{1}}}{\frac{\sum_{\text{i}=\text{1}}^{\text{k}}\sum_{\text{j}=\text{1}}^{{\text{n}}_{\text{i}}}{\left(X_{ij}-\overset{―}{X_{i}}\right)}^{\text{2}}}{\text{N}-\text{k}}} \end{array}$$ (9)

Between-group variance reflects the degree of mean difference across categories, while the within-group variance captures the variability of samples within each category. A higher F-value indicates that the feature shows stronger discriminative power among different classes and exerts a greater influence on the target variable. Therefore, such features are considered important and retained. Through F-test, key drug features highly correlated with the target variable can be selected, leading to an optimized model structure with improved prediction accuracy and generalization capability.

### Construction of the ensemble model

To effectively capture the potential complex association patterns between drugs and diseases, this study proposes a classification framework based on ensemble learning, which integrates three classical four-based models: Extreme Gradient Boosting (XGBoost), Decision Tree, Random Forest and HyperFast. The objective of this ensemble strategy is to combine the structural advantages of different models to improve the overall prediction accuracy and generalization capability.

### Extreme Gradient Boosting (XGBoost)

XGBoost (Extreme Gradient Boosting) is an advanced implementation of the Gradient Boosting Decision Tree (GBDT) framework, widely applied to classification and regression tasks on structured data due to its high efficiency, flexibility, and excellent scalability. By iteratively minimizing a loss function and introducing a regularization term, XGBoost effectively controls model complexity and reduces the risk of overfitting. This process allows it to leverage precise feature split gains to deeply explore non-linear relationships and complex interaction patterns among high-dimensional features. In this study, XGBoost takes the feature vectors of drugs and diseases as input. Leveraging its tree structure’s automatic splitting mechanism, it deeply investigates the complex connections among high-dimensional features, thereby implicitly identifying the most critical ones. Thanks to its strong non-linear modeling capabilities and its native mechanism for handling missing values, XGBoost demonstrates excellent performance and robustness when modeling the intricate interactions between drugs and diseases.

### Decision tree

The Decision Tree, characterized by its intuitive structure and high interpretability, is widely applied to both classification and regression tasks. It captures relationships between features by recursively partitioning the dataset into subsets, building a tree-like model according to criteria such as Gini impurity or information gain. This makes it suitable for solving a wide range of structured data problems. In this study, a Decision Tree with fixed hyperparameter settings is employed to jointly model the 300-dimensional drug features and 64-dimensional disease features. The model creates a series of decision rules in the feature space based on feature splits, automatically learning the relationships between drug and disease features. It selects the optimal feature and performs node splitting according to the Gini impurity. After training, the Decision Tree can make step-by-step decisions based on the input drug-disease feature vector, ultimately assigning a corresponding class label. It also provides a complete tree structure including tree depth, number of leaf nodes, and feature importance, serving as a foundation for subsequent interpretability analysis.

### Random forest

Random Forest is a classic Bagging-based ensemble learning method, which constructs multiple independent decision trees and aggregates their predictions to maintain high classification accuracy while effectively suppressing overfitting. By leveraging the Bagging technique, Random Forest performs random sampling with replacement on the training dataset to build diverse decision trees, thereby enhancing generalization performance. During the construction of each tree, Random Forest randomly selects a subset of features at each node split, ensuring independence among trees. The final prediction is made by majority voting or averaging across all trees. In this study, the Random Forest model is implemented by randomly sampling with replacement from the original dataset to create multiple training sets, ensuring data diversity across trees. At each split node, a random subset of features is considered for splitting, which effectively reduces the risk of overfitting and improves the model’s generalization ability. By capturing various data patterns and complex nonlinear relationships, Random Forest enhances the classification performance through the ensemble of multiple tree predictions [44].

### HyperFast

**Fig 3:**
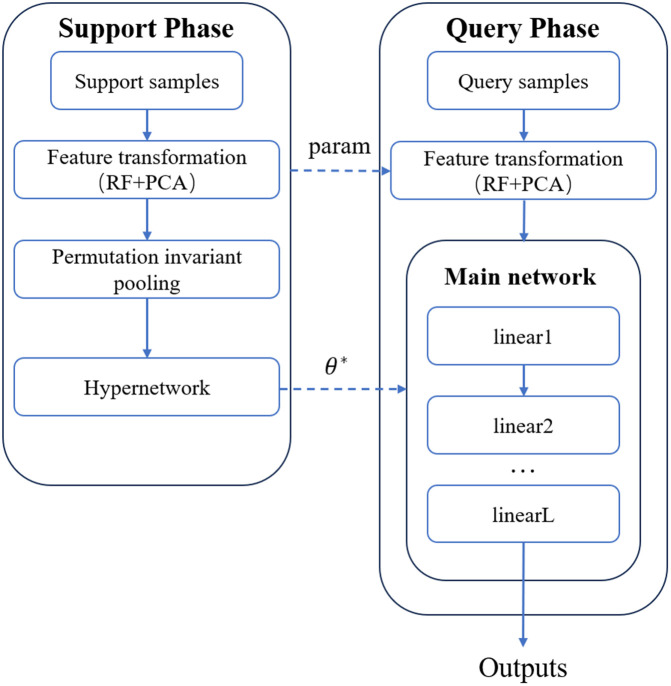
The framework of HyperFast.

HyperFast [45] is constructed based on a hierarchical mapping mechanism involving a Hypernetwork and a Main Network. The overall framework consists of three core modules: “Feature Preprocessing and Mapping,” “Hypernetwork Weight Generation,” and “Main Network Classification Inference,” forming an efficient one-shot generative classification system. The specific architecture is shown in Fig 3.

In the feature preprocessing phase, the input sample matrix is denoted as:


$$\begin{array}{c} X=[x_{\text{1}},x_{\text{2}},\ldots ,x_{n}{]}^{⊤}\in R^{n\times d} \end{array}$$ (10)

where *n* is the number of samples and *d* is the feature dimension. The model first performs a standardization mapping on the input:


$$\begin{array}{c} \tilde{X}=\frac{X-\mu }{\sigma } \end{array}$$ (11)

where μ and σ are the mean and standard deviation of the feature dimensions, respectively. Subsequently, HyperFast introduces random feature transformation to approximate nonlinear kernel mapping. Through a Gaussian random matrix $R\in R^{d\times h}$ and a bias term b, the model maps the input to a high-dimensional random feature space:


$$\begin{array}{c} Z=\phi \left(\tilde{X}R+b\right) \end{array}$$ (12)

where ϕ(·) is a nonlinear activation function (such as ReLU). This process mathematically approximates the implicit projection of an arc-cosine kernel. To further enhance feature compactness and stability, the model performs Principal Component Analysis (PCA) on the random feature space, retaining the top k principal components:


$$\begin{array}{c} {\text{Z}}^{\prime }=\;\text{Z}\;W_{k} \end{array}$$ (13)

where $W_{k}\in R^{h\times k}$ is the projection matrix obtained from the eigendecomposition of the feature covariance matrix. This step effectively reduces feature redundancy and noise interference while preserving principal information.

In the weight generation phase, the hypernetwork $H_{\theta }$ receives the support set representation $Z_{S}^{\prime }$ and its labels $Y_{S}$, generating all parameters for the main network via a single forward pass:


$$\begin{array}{c} W=H_{\theta }\left(Z_{S}^{\prime },Y_{S}\right) \end{array}$$ (14)

where $W=\{W^{\left(\text{1}\right)},W^{\left(\text{2}\right)},\ldots ,W^{\left(L\right)}\}$ represents the set of hierarchical weights for the main network. The hypernetwork adopts a layer-wise weight generation strategy: for the first L-1 layers, it utilizes global average pooling for feature statistic aggregation; for the classification layer (the L-th layer),it combines class-wise mean pooling and bias adjustment to generate class-discriminative vectors. This mechanism enables the model to generate task-specific parameters directly based on the feature distribution of support samples, achieving rapid modeling without iterative training.

The classification inference phase is performed by the main network $f_{W}(\cdot )$, which is structured as a three-layer Multi-Layer Perceptron (MLP) and introduces residual connections between layers to improve gradient stability and representational capacity. For query set samples $Z_{Q}^{\prime }$, the model performs forward propagation to obtain class predictions:


$$\begin{array}{c} \hat{y}=f_{W}\left(Z_{Q}^{\prime }\right) \end{array}$$ (15)

and obtains the posterior probability of the target class via the Softmax function:


$$\begin{array}{c} p\left(y=\text{1}|Z_{Q}^{\prime }\right)=\frac{\text{exp}\left({\hat{y}}_{\text{1}}\right)}{\text{exp}\left({\hat{y}}_{\text{0}}\right)+\text{exp}\left({\hat{y}}_{\text{1}}\right)} \end{array}$$ (16)

The entire inference process is completed under the static weights generated by the hypernetwork, without parameter updates or gradient backpropagation, thereby significantly enhancing inference efficiency and task adaptability. In summary, the HyperFast classifier achieves an end-to-end mapping from input features to prediction results through random feature kernel mapping, hierarchical weight generation, and lightweight forward inference. Its core innovation lies in replacing the traditional training process with a hypernetwork, realizing efficient non-iterative classification via one-shot weight generation, endowing the model with outstanding generalization performance and computational scalability across different tasks.

To fully leverage the complementary advantages of different models in feature expression and generalization capability, this study adopts a two-layer Stacking ensemble strategy to realize multi-model fusion. This method introduces four types of heterogeneous learners at the Level-0 layer—XGBoost, Decision Tree, Random Forest, and HyperFast—and obtains Out-of-Fold (OOF) prediction results through $K$-fold cross-validation to provide robust inputs for upper-level learning. Subsequently, the Level-1 layer employs Logistic Regression as the meta-model, using the OOF prediction probability matrix as features to learn the optimal combination of outputs from different base models, thereby statistically achieving adaptive weighting and nonlinear fusion. Overall, this Stacking strategy learns the complementary relationships of different models at the meta-level, making the fused model superior to any single model in both prediction accuracy and stability. Experimental results indicate that this method can effectively capture multimodal structural features in drug–disease association tasks, providing an efficient and generalizable systemic solution. Model parameter configurations are shown in Table 1. It is worth noting that as a pre-trained hypernetwork, HyperFast’s parameters are frozen during the meta-training phase, relying on rapid weight generation to achieve task adaptability, requiring no gradient updates or hyperparameter tuning during integration.

**Table 1:** Parameter settings of individual models.

Model	Parameter	Value
XGBoost	colsample_bytree	0.8
	subsample	0.8
	learning_rate	0.1
	max_depth	5
	n_estimators	100
	random_state	42
DecisionTree	min_samples_leaf	5
	min_samples_split	10
	max_depth	None
	random_state	42
RandomForest	min_samples_leaf	10
	min_samples_split	15
	max_depth	5
	n_estimators	30
	random_state	42

## Results and discussion

### Evaluation metrics

The predictive performance of the model was evaluated using several standard metrics: Accuracy (ACC), Precision (Pre), Sensitivity (SN), Specificity (SP), Matthews Correlation Coefficient (MCC), Area Under the Receiver Operating Characteristic Curve (AUC), and Area Under the Precision-Recall Curve (AUPR) [46–50]. The mathematical definitions for these metrics are provided in Equations (17)–(23) [51]. Specifically, in the context of drug-disease association prediction, a sample is defined as a positive sample if it represents a verified association; otherwise, it is classified as a negative sample.


$$\begin{array}{c} ACC=\frac{TP+TN}{TP+TN+FP+FN} \end{array}$$ (17)

$$\begin{array}{c} Pre=\frac{TP}{TP+FP} \end{array}$$ (18)

$$\begin{array}{c} SN=\frac{TP}{TP+FN} \end{array}$$ (19)

$$\begin{array}{c} SP=\frac{TN}{TN+FP} \end{array}$$ (20)

$$\begin{array}{c} MCC=\frac{\left(TP\times TN)-(FN\times FP\right)}{\sqrt{\left(TP+FN)\times (TN+FP)\times (TP+FP)\times (TN+FN\right)}} \end{array}$$ (21)

TP represents the number of verified associations correctly predicted as positive; TN is the number of non-associations correctly predicted as negative; FP is the number of non-associations incorrectly predicted as positive; and FN is the number of verified associations incorrectly predicted as negative.


$$\begin{array}{c} AUC=\frac{\sum_{i=\text{1}}^{n}T_{i}}{nT} \end{array}$$ (22)

The Receiver Operating Characteristic (ROC) curve is an essential tool for evaluating the discriminative ability of predictive models. Its horizontal axis represents sensitivity, while the vertical axis corresponds to specificity. By plotting this curve, the model’s performance across different threshold values can be visualized. The area under the ROC curve (AUC) serves as a core indicator, ranging between 0 and 1. A higher AUC value indicates superior model discrimination. Specifically, an AUC close to 1 suggests near-perfect separation between positive and negative samples, while an AUC of 0.5 implies performance equivalent to random guessing.


$$AUPR=\sum_{k=\text{2}}^{n}\frac{\left(R_{k}-R_{k-\text{1}}\right)\cdot \left(P_{k}+P_{k-\text{1}}\right)}{\text{2}}$$ (23)

The Area Under the Precision-Recall Curve (AUPR) is another important evaluation metric in machine learning. It is calculated by integrating the area under the Precision-Recall curve, providing a comprehensive measure of the classifier’s performance across different decision thresholds. The value of AUPR also ranges from 0 to 1, with a value closer to 1 indicating superior discrimination ability and predictive stability. Compared to other metrics, AUPR is particularly well-suited for scenarios with imbalanced class distributions, as it more accurately evaluates a model’s effectiveness in identifying the minority class.

### Effectiveness of drug feature extraction

Accurate drug feature extraction serves as the cornerstone of successful Drug-Disease Association (DDA) prediction. High-quality embeddings enable learning algorithms to discriminate subtle variations in molecular function, enhance generalization to novel compounds, and ultimately improve prediction reliability. To rigorously validate the rationale underpinning our selected feature extraction framework, we conducted a comparative analysis involving five distinct drug embedding strategies. The results are presented in Table 2.

**Table 2:** Comparison of molecular embeddings.

Method	Accuracy	AUC	AUPR	Precision	Recall	F1-score	MCC	Sensitivity
**Mol2vec**	0.9009	0.9585	0.9656	0.9432	0.8556	0.8973	0.8055	0.8556
**K-BERT**	0.9023	0.9596	0.9660	0.9444	0.8572	0.8987	0.8082	0.8572
**GIN**	0.8928	0.9407	0.9498	0.9386	0.8383	0.8856	0.7897	0.8383
**Chemberta-2**	0.9009	0.9578	0.9642	0.9385	0.8604	0.8978	0.8048	0.8604
**Mol2vec+K-BERT**	0.9305	0.9725	0.9780	0.9591	0.9035	0.9305	0.8627	0.9035

Our experimental results indicate that while single-feature methods such as Mol2vec, K-BERT, GIN, and ChemBERTa-2 each capture distinct aspects of molecular information, the variances in their performance are only moderate. In sharp contrast, our proposed fusion strategy (Mol2vec + K-BERT) demonstrates a significant performance leap, achieving the highest scores across all eight evaluation metrics, with an AUC of 0.9725 and an F1-score of 0.9305. This superiority validates the complementarity hypothesis of our study: Mol2vec explicitly encodes the frequency of local chemical substructures, whereas K-BERT injects global semantics and domain knowledge. By integrating these two dimensions, the model overcomes the limitations inherent in unimodal approaches, thereby providing a robust and comprehensive input for downstream predictors.

### Effectiveness of undersampling algorithms

**Fig 4:**
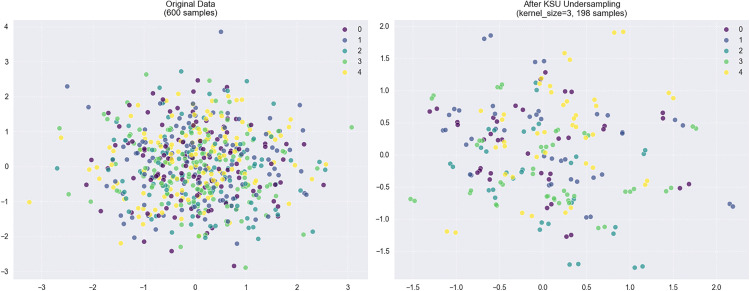
Comparison of data distribution before and after applying KSU undersampling.

Due to the inherent class imbalance in drug–disease association datasets, the scarcity of positive samples often leads to models that underestimate their misclassification risk. This results in reduced sensitivity and inflated specificity. To mitigate this bias, this study introduces undersampling algorithms to balance the distribution of positive and negative samples. The KSU algorithm, in particular, effectively reduces the number of samples in the majority class, creating a more balanced dataset for model training, as visually demonstrated in Fig 4.

By comparing the AUC performance of several algorithms (including KSU, NCR, NearMiss, OSS, and Random Undersampling), the KSU algorithm was ultimately selected. Moreover, we further optimized its sample selection strategy by tailoring the distance metrics according to different model architectures. The experiments cover a wide range of machine learning models (such as XGBoost, Random Forest, Naive Bayes, etc.) as well as deep learning models (including DNN, BiLSTM, GRU, etc.). The AUC performance of each model under different undersampling algorithms is summarized in Table 3. For this initial benchmark comparison, the KSU algorithm utilized the standard Euclidean Distance as its metric to ensure a fair and consistent baseline.

**Table 3:** AUC comparison of different models using various undersampling algorithms (KSU with Euclidean Distance).

Model	Undersampling Algorithms				
	KSU	NCR	NearMiss	OSS	Random
**XGBoost**	0.9623	0.8330	0.9523	0.8295	0.8360
**RandomForest**	0.9208	0.7293	0.8842	0.7213	0.7299
**Naive_bayes**	0.6692	0.5976	0.6459	0.5910	0.5966
**DecisionTree**	0.9208	0.6812	0.8580	0.6758	0.6964
**DNN**	0.9189	0.7636	0.8671	0.7610	0.7625
**BiLSTM**	0.8790	0.7469	0.8314	0.7350	0.7450
**GRU**	0.8689	0.7364	0.8160	0.7319	0.7364
**MLP**	0.8942	0.7657	0.8568	0.7582	0.7622
**RNN**	0.7667	0.6539	0.7206	0.6575	0.6700
**TextRCNN**	0.9073	0.7628	0.8538	0.7557	0.7602

The results indicate that the KSU undersampling algorithm achieved the optimal performance across all models. Specifically, XGBoost reached an AUC of 0.9623, RandomForest achieved 0.9208, DecisionTree also reached 0.9208, and DNN attained an AUC of 0.9189. To further optimize the sample selection strategy and investigate the interaction between model architectures and distance metrics, we evaluated the performance of six distinct classifiers—XGBoost, Decision Tree, Random Forest, DNN, TextRCNN, and RNN—using five different distance formulas within the K-means undersampling (KSU) framework. The comparative AUC scores are presented in Table 4.

**Table 4:** Comparison of AUC scores for different models using various distance formulas.

Model	Distance Formula				
	Hamming Distance	Manhattan Distance	Minkowski Distance	Euclidean Distance	Chebyshev Distance
**XGBoost**	0.9696	0.9597	0.9637	0.9626	0.9527
**DecisionTree**	0.9273	0.9186	0.9187	0.9205	0.9128
**RandomForest**	0.9264	0.9190	0.9217	0.9207	0.9220
**DNN**	0.8918	0.9183	0.9108	0.9181	0.9120
**TextRCNN**	0.8699	0.9046	0.9101	0.9082	0.9043
**RNN**	0.7358	0.7680	0.7833	0.7667	0.8007

The results reveal a clear architectural divergence regarding metric sensitivity. Tree-based models (XGBoost, Decision Tree, Random Forest) consistently achieved optimal performance with Hamming Distance, likely due to its alignment with the discrete nature of molecular fingerprints. In contrast, sequence-based and deep models favored spatial metrics: notably, the RNN peaked using Chebyshev Distance (AUC 0.8007) but performed poorly with Hamming (0.7358), while TextRCNN preferred Minkowski Distance (0.9101). Despite these variations, the combination of XGBoost and Hamming Distance yielded the superior overall AUC (0.9696). Consequently, Hamming Distance was prioritized for the final prediction framework. Furthermore, to quantify the joint effect of undersampling and distance metrics, the performance of the ensemble model before and after sampling was compared, as shown in Table 5.

**Table 5:** Comparison of model performance before and after training set sampling and with different distance formulas.

	Accuracy	AUC	F1-score	MCC	Sensitivity
**Before sampling**	0.8913	0.7593	0.1489	0.2193	0.0832
**After sampling (Euclidean Distance)**	0.9117	0.91173	0.9046	0.8326	0.8376
**After sampling (Optimal - Hamming Distance)**	0.9395	0.9395	0.9374	0.8811	0.9053

As indicated by the data in the table, the model’s performance improved significantly across multiple metrics after sampling. Prior to sampling, the model exhibited an Accuracy of 0.8913, an AUC of 0.7593, an F1-score of 0.1489, an MCC of 0.2193, and a Sensitivity of just 0.0832. These results indicate a substantial bias on the class-imbalanced dataset and a particularly weak ability to identify positive class samples. After applying undersampling with the baseline Euclidean Distance, the model’s performance improved significantly across all metrics. Notably, when the optimal Hamming Distance identified in Table 5 was applied, performance was further boosted, with sensitivity increasing to 0.9053, the F1 score reaching 0.9374, and the MCC improving to 0.8811. These results indicate substantial enhancement in the model’s ability to predict both positive and negative classes, especially in identifying positive samples (sensitivity) and achieving overall balanced performance (F1 score). Therefore, both undersampling and the selection of an appropriate distance metric play a critical role in boosting model performance, particularly in handling imbalanced datasets.

### Effectiveness of F-TEST feature selection

**Fig 5:**
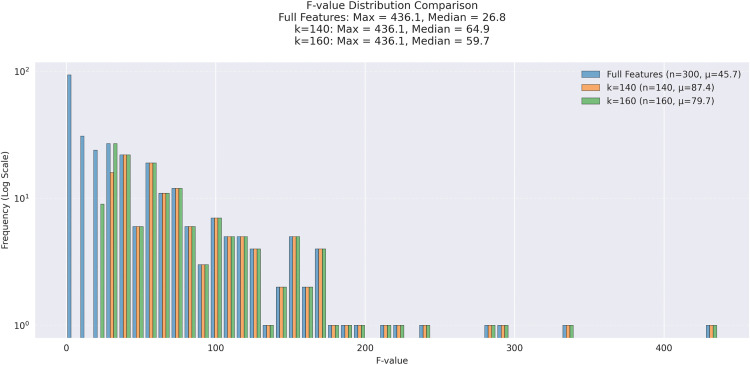
Comparison of F values for different numbers of features.

To evaluate the effectiveness of the F-test feature selection method in this study, we performed dimensionality reduction on the original features and plotted the distribution of F-values for different dimensions, as shown in Fig 5. The figure displays the frequency distribution (on a logarithmic scale) of F-values for the complete feature set (n = 300) as well as for feature subsets reduced to 140 and 160 dimensions. It can be observed that as the number of features decreases, the distribution of F-values shifts toward higher-value regions. In particular, the proportion of high F-value features (e.g., F > 100) significantly increases in the k = 140 and k = 160 subsets, indicating that the retained features exhibit stronger discriminative power between classes. Meanwhile, the number of low F-value features drops sharply, suggesting that redundant or noisy features were effectively removed. Additionally, the statistical information in the legend confirms that the discriminative capability of the feature sets was enhanced through dimensionality reduction. The average F-value of the full feature set was 45.7, which increased to 87.4 when the top 140 features were selected and remained high at 79.7 for 160 features. These results demonstrate that the F-test not only enables effective dimensionality reduction but also serves as a robust feature selection method, contributing to improved model training efficiency and predictive performance.

**Fig 6:**
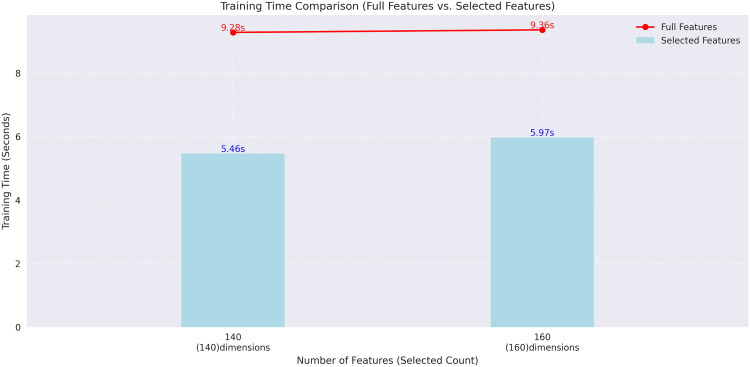
Comparison of model training time under different feature dimensions.

To further assess the effectiveness of the F-test in improving model training efficiency, we conducted a comparative analysis of training time under different drug feature dimensions, as illustrated in Fig 6. When the number of features was reduced to 140 and 160, the training time decreased by 3.82 seconds and 3.39 seconds, respectively, indicating a significant improvement in computational performance due to dimensionality reduction. At the same time, model accuracy remained stable or even slightly improved on these feature subsets, suggesting that dimensionality reduction did not impair predictive performance but rather enhanced the model’s generalization capability. Therefore, the F-test method effectively compresses the high-dimensional drug feature space in this task, selecting a subset of features with stronger discriminatory power. This approach substantially reduces computational overhead while maintaining predictive performance, proving it to be an efficient and robust feature selection strategy.

### Effectiveness of the ensemble model

This study evaluated the performance of 15 mainstream machine learning and deep learning classification models through a comparative experiment to systematically select base models. Recognizing that the performance of each model is highly dependent on the selected parameters, hyperparameter tuning was conducted via grid search to identify the optimal parameter subset for each model. To avoid overfitting and ensure the reliability of the results, ten-fold cross-validation was used in conjunction with grid search. Based on the results obtained from the grid search, a model with the optimal parameter set was constructed for each algorithm. Subsequently, the best model was selected from this set based on its predictive performance on the validation set. The experimental results are shown in Table 6.

**Table 6:** Comparison results of models.

Name	Accuracy	AUC	AUPR	Precision	Recall	F1-score	MCC	Sensitivity
**XGBoost**	0.9121	0.9623	0.9710	0.9843	0.8376	0.9050	0.8336	0.8376
**SVM**	0.7757	0.8542	0.8738	0.8057	0.7269	0.7642	0.5542	0.7269
**RandomForest**	0.8852	0.9208	0.9433	0.9723	0.7930	0.8735	0.7838	0.7930
**naive_bayes**	0.5988	0.6692	0.6812	0.5741	0.7680	0.6569	0.2100	0.7680
**DecisionTree**	0.8901	0.9208	0.9438	0.9539	0.8199	0.8818	0.7881	0.8199
**TextRCNN**	0.8209	0.9073	0.9215	0.8316	0.8104	0.8192	0.6445	0.8104
**RNN**	0.7110	0.7667	0.7477	0.7256	0.7037	0.7074	0.4311	0.7037
**MLP**	0.8023	0.8942	0.9077	0.8430	0.7546	0.7903	0.6146	0.7546
**Logistics**	0.7751	0.8545	0.8723	0.8017	0.7310	0.7647	0.5523	0.7310
**LDA**	0.7716	0.8502	0.8636	0.7997	0.7248	0.7603	0.5455	0.7248
**GRU**	0.7853	0.8689	0.8826	0.8185	0.7435	0.7758	0.5776	0.7435
**DNN**	0.8428	0.9189	0.9325	0.8875	0.7869	0.8333	0.6911	0.7869
**BiLSTM**	0.7864	0.8790	0.8930	0.8188	0.7516	0.7761	0.5840	0.7516
**AttentionLSTM**	0.7818	0.8634	0.8744	0.8223	0.7260	0.7682	0.5708	0.7260
**HyperFast**	0.8648	0.9401	0.9479	0.8951	0.8322	0.8625	0.7318	0.8322

Based on the experimental results after hyperparameter optimization via ten-fold cross-validation and grid search, quantitative analysis indicates that XGBoost, HyperFast, Decision Tree, and Random Forest form a distinct “first tier” across key performance metrics, significantly outperforming other baseline models. This conclusion is strongly supported by the data: XGBoost leads in all evaluation metrics, achieving the best performance particularly in AUC (0.9623), AUPR (0.9710), and F1-score (0.9050). HyperFast, as a novel meta-learning model, demonstrates exceptional competitiveness, ranking second in both AUC (0.9401) and AUPR (0.9479), surpassing all traditional models except XGBoost. Meanwhile, Decision Tree (F1: 0.8818) and Random Forest (F1: 0.8735) closely follow in accuracy and F1 scores, ranking among the top performers.

To obtain the most robust predictive performance, this study does not rely on a single model but instead selects these four models—which possess advantageous and highly complementary performance and mechanisms—to jointly construct the Level-0 base model layer of the Stacking ensemble.Specifically: XGBoost (AUC 0.9623), as a representative of gradient boosting, provides extremely high prediction accuracy and overfitting control by integrating weak learners; HyperFast (AUC 0.9401) represents a novel meta-learning paradigm, where its “one-shot inference modeling” mechanism offers a unique perspective for model combination, demonstrating superior performance in key metrics; Random Forest (AUC 0.9208) adopts a Bagging strategy, enhancing model stability and generalization capability through the introduction of randomness; and Decision Tree (AUC 0.9208) provides transparent decision paths via Gini impurity splitting rules, enhancing model interpretability.

**Fig 7:**
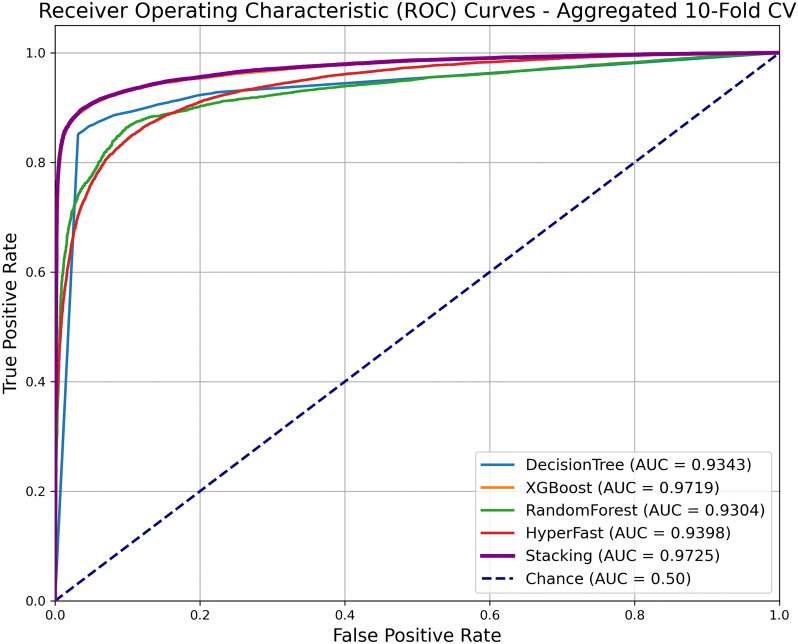
ROC curves of the individual models and the ensemble model.

The ROC curves for each model and the final fusion model are shown in Fig 7. As seen in the figure, the AUC values of all four single base models (XGBoost, HyperFast, Decision Tree, Random Forest) are lower than that of the final fusion model. This indicates that the Level-1 meta-model (Logistic Regression) effectively learns and integrates the strengths of these four heterogeneous base learners, mitigating potential bias and variance issues inherent in single models. By intelligently weighting the predicted probabilities from the Level-0 models, the fusion model significantly improves classification accuracy and stability. When handling tasks such as drug–disease association prediction—which are high-dimensional, complex, and potentially subject to data imbalance or noise—the fusion method demonstrates stronger generalization capability and reduces the risk of overfitting, thus yielding an AUC value higher than any single model. This result powerfully validates the effectiveness of the proposed four-model fusion strategy.

### Analysis of model complexity and performance trade-off

While pursuing high prediction accuracy, the computational complexity of a model is a critical factor determining its practicality and scalability. The final prediction model adopted in this study is based on a weighted Ensemble Model, combining the complementary advantages of four heterogeneous base learners: XGBoost, RandomForest, DecisionTree, and HyperFast. Therefore, quantitatively analyzing the overall computational cost of this ensemble model and comparing it with its independent components is of great significance for model selection under different computational environments. This section provides a comprehensive evaluation of the performance and complexity of each model based on training duration and peak memory usage. It should be noted that the total training time of the ensemble model is calculated as the sum of the training durations of its constituent base learners. Relevant results are presented in Table 7.

**Table 7:** Comprehensive comparison of performance and complexity between the final ensemble model and its components.

Model	AUC	Training time, seconds	Peak Memory Usage, MB
**FKSUDDAPre**	0.9725	109.1991	64.0186
**XGBoost**	0.9730	2.8627	0.3031
**RandomForest**	0.9342	2.1108	3.0191
**DecisionTree**	0.9306	4.8928	1.4732
**HyperFast**	0.9401	99.3328	64.0186

As indicated by the data analysis in Table 7, FKSUDDAPre achieved highly competitive predictive performance (AUC = 0.9725), attributable to its integration of base models with diverse mechanisms. However, this performance improvement is accompanied by increased computational costs. The total training duration (109.20 seconds) and peak memory usage (64.02 MB) of the ensemble model are primarily dominated by the HyperFast component (99.33 seconds, 64.02 MB). Although HyperFast, as a meta-learning model, introduces higher resource overhead, its unique “one-shot inference” mechanism provides the ensemble framework with a perspective distinct from traditional tree models, enhancing the model’s generalization potential under complex distributions.

In contrast, the traditional tree model components demonstrated extremely high computational efficiency: RandomForest emerged as the preferred choice for rapid iteration with the shortest training time of 2.11 seconds; XGBoost stood out most prominently among the standalone models, not only boasting fast computation (2.86 seconds) but also performing on par with, or even slightly better than, the ensemble model in terms of AUC (0.9730). This indicates that in scenarios where resources are severely constrained and deploying the full ensemble model is unfeasible, XGBoost serves as the optimal standalone alternative.

### Comparison with other advanced methods

To further evaluate the effectiveness of the proposed method, we conducted a comparative study on the same dataset with several state-of-the-art approaches, including HINGRL [28](2021), CMFMTL [18](2020), HDGAT [52](2024), GRLGB [53](2023), MFFGCN [8](2023), and AMDGT [30](2023). All of these methods utilized publicly available datasets as reported in their original publications.

**Fig 8:**
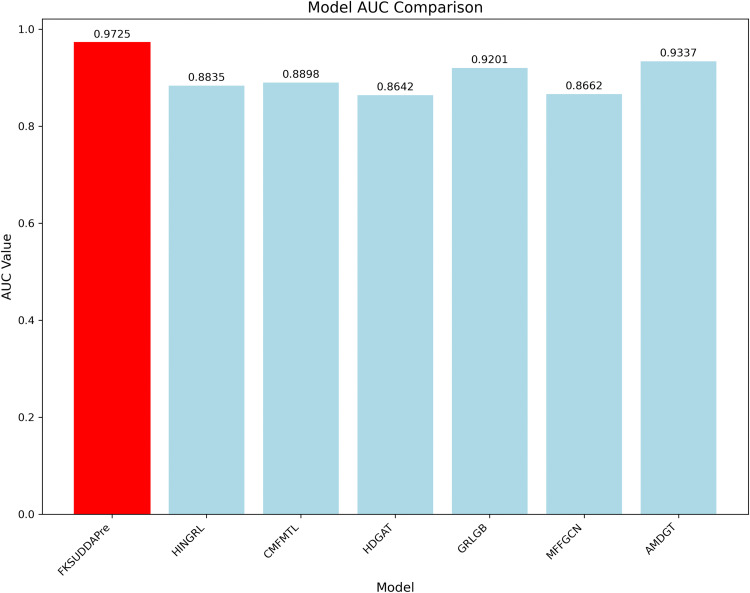
Comparison of AUC values between FKSUDDAPre and other existing drug–disease interaction prediction methods.

Fig 8 presents the comparison of the average AUC scores achieved by each method on the B dataset. The results clearly show that our method outperformed all other approaches, achieving an average AUC of 0.9725. Specifically, it surpassed the comparative methods by 8.9%, 8.27%, 10.83%, 5.24%, 10.63%, and 3.88%, respectively. These findings strongly demonstrate the superior performance and robustness of our proposed approach.

### LIME interpretability analysis

Although deep learning models demonstrate exceptional performance on complex tasks, their nonlinear structures and high-dimensional parameter spaces make their prediction mechanisms difficult to interpret directly. To enhance the transparency of the model’s decision-making process, this study adopted LIME (Local Interpretable Model-agnostic Explanations) as a tool for local interpretability analysis [54]. The fundamental idea of LIME is to generate a series of perturbed samples in the neighborhood of an original input sample. It then obtains the predictions for these perturbed samples from the black-box model and uses them to fit a weighted linear model. This process approximates the model’s true behavior in the local space, explaining the marginal impact of features on an individual prediction. This method is model-agnostic, offering broad applicability and strong human readability.

**Fig 9:**
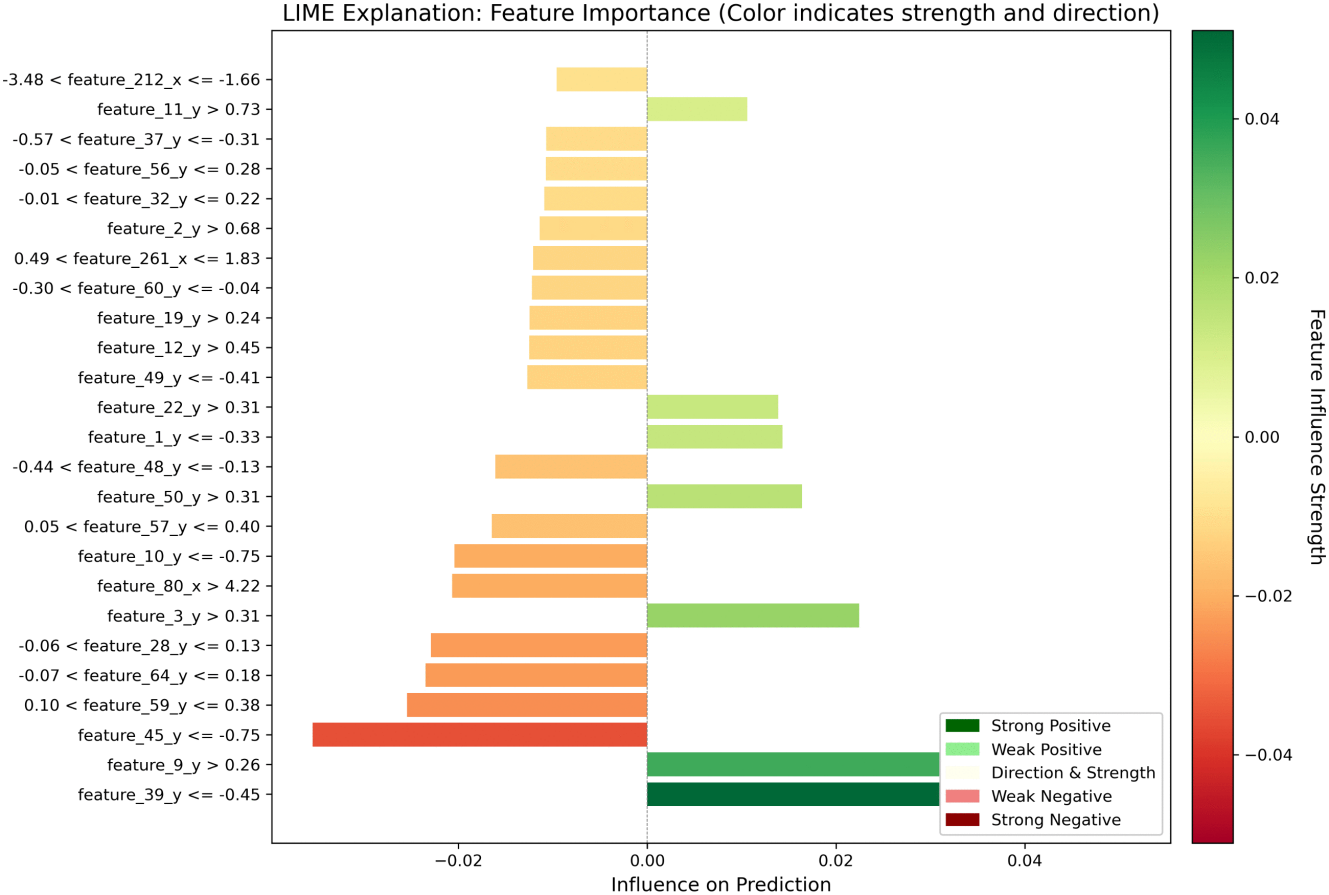
LIME-based feature importance analysis.

Fig 9 presents a LIME-based feature importance visualization for a specific test sample, aiming to illustrate the local influence of input features on the model output during prediction. The horizontal axis represents the influence on prediction, while the vertical axis lists the top 25 key features sorted by importance, each annotated with its corresponding threshold condition (e.g., “feature_44_y ≤ -0.30”). This effectively approximates the model’s local decision rule. The color coding employs a gradient heatmap, where deep green indicates that a feature’s current value makes a significant positive contribution to the positive class prediction. In contrast, deep red signifies a strong inhibitory effect (negative contribution) on the positive class prediction. Lighter shades indicate a weaker influence.

From the figure, features such as feature_44_y ≤ -0.30, feature_63_y ≤ -0.40, and feature_18_y ≤ -0.33 appear in dark green, indicating that they are major positive drivers for the model’s prediction of the positive class in this sample. Conversely, feature_34_y > 0.32, feature_33_y > 0.37, and feature_41_y > 0.20 are shown in dark red, suggesting they strongly suppress the model’s positive class prediction, functioning as negative indicators in the local decision logic. Each feature’s logical expression includes a specific threshold boundary, offering insight into the local decision boundary around the sample. This enables the behavior of the complex model near this sample point to be approximated in a rule-based manner, improving interpretability.

### Case study

To evaluate the practical applicability of the proposed model (FKSUDDAPre), we conducted an in-depth case study on two representative neurodegenerative diseases: Alzheimer’s Disease (AD) and Parkinson’s Disease (PD). All known drug-disease associations in the dataset were used as training data, and the model was trained to predict the association probabilities for all unknown drug-disease pairs. Subsequently, drugs were ranked based on the predicted probabilities, and the top 10 candidate drugs were selected for each disease.

Alzheimer’s Disease is the most common form of progressive dementia in the elderly and represents a severe neurodegenerative condition. Table 8 presents the top 10 candidate drugs predicted by the model for AD treatment. Among them, 8 drugs have been validated by existing literature, indicating the model’s strong practical value. For example, Tolbutamide, a KATP modulator, was found to provide significant protection against Aβ-induced memory defects. Albendazole, an FDA-approved anti-parasitic drug, has been shown in recent studies to reduce levels of AD-associated Tau protein in cells. Furthermore, Felodipine, an L-type calcium channel blocker, can induce autophagy and clear various aggregation-prone proteins related to neurodegenerative diseases.

**Table 8:** The top 10 potential drugs identified for Alzheimer’s disease.

Rank	drug id	Drug name	Score	Evidence (PMID)
1	DB01124	Tolbutamide	0.9909	28222502
2	DB01435	Antipyrine	0.9897	25847999
3	DB00518	Albendazole	0.9895	N/A
4	DB01400	Neostigmine	0.9895	N/A
5	DB01023	Felodipine	0.9892	31000720
6	DB00788	Naproxen	0.9891	17460158
7	DB00672	Chlorpropamide	0.9886	39535041
8	DB00201	Caffeine	0.9885	37371547
9	DB00549	Zafirlukast	0.9883	34479635
10	DB01595	Nitrazepam	0.9883	25208536

Parkinson’s Disease (PD) is the second most common neurodegenerative disorder after Alzheimer’s Disease. Table 9 lists the top 10 predicted candidate drugs for PD generated by the model. Among them, 6 drugs have been previously reported in relevant literature, further demonstrating the model’s effectiveness in identifying potential therapeutic agents. For example, Caffeine is supported by numerous epidemiological studies linking its intake to a reduced risk of developing PD. Albendazole has demonstrated neuroprotective effects in PD models by activating key pathways like Nurr1 and suppressing neuroinflammation. Furthermore, Chlorpropamide has been shown to inhibit the fibrillization of alpha-synuclein, a key pathological process in PD.

**Table 9:** The top 10 potential drugs identified for Parkinson’s disease.

Rank	drug id	Drug name	Score	Evidence
1	DB01124	Tolbutamide	0.9909	34380882
2	DB00281	Lidocaine	0.9897	N/A
3	DB01435	Antipyrine	0.9897	N/A
4	DB00518	Albendazole	0.9895	31408200
5	DB01400	Neostigmine	0.9895	37546148
6	DB00672	Chlorpropamide	0.9886	37546148
7	DB00201	Caffeine	0.9885	33390888
8	DB00549	Zafirlukast	0.9883	N/A
9	DB01595	Nitrazepam	0.9883	N/A
10	DB01054	Nitrendipine	0.9882	24910980

These findings highlight the practical applicability and clinical relevance of the FKSUDDAPre model in drug repurposing for neurodegenerative diseases. By accurately prioritizing candidate drugs with known or emerging therapeutic relevance, the model can serve as a valuable decision-support tool for clinicians and biomedical researchers. For Alzheimer’s Disease and Parkinson’s Disease—both lacking effective disease-modifying treatments—FKSUDDAPre offers a systematic approach to rapidly identify repurposable compounds from existing drug libraries, potentially accelerating the drug discovery process and reducing clinical trial costs. Moreover, the inclusion of non-traditional drug classes (e.g., antihistamines and chemotherapeutic agents) among the top candidates may stimulate new clinical hypotheses and broaden the scope of pharmacological intervention strategies. Future work integrating this predictive framework with clinical trial design and patient stratification may further enhance its translational impact.

## Conclusion

In this study, we proposed a novel drug–disease association prediction model named FKSUDDAPre. Specifically, we first constructed integrated feature representations for drugs and diseases using a hybrid feature extraction strategy combining an ensemble of Mol2vec and K-BERT with DeepWalk. To address the class imbalance problem, we designed a balanced dataset based on the AMDKSU algorithm, which combines adaptive multi-distance metrics with dynamic group-based sampling strategies. During the feature selection stage, the F-test method was employed to extract a highly discriminative subset from the high-dimensional fused features, thereby improving computational efficiency and model generalization. For classification, we constructed an ensemble model by integrating XGBoost, Decision Tree, Random Forest, and HyperFast, leveraging the nonlinear modeling capability of gradient boosting, the interpretability of tree-based models, the overfitting resistance of bagging, and the rapid, meta-learning-based inference of HyperFast. We systematically evaluated the performance of each base model and the ensemble model, and further validated the effectiveness of AMDKSU and F-test in class balancing and feature selection. In addition, model interpretability was enhanced using LIME-based local analysis, and the practical potential of the model was demonstrated through two case studies on Alzheimer’s disease (AD) and Parkinson’s disease (PD), identifying the top 10 candidate drugs for each condition.

The experimental results demonstrate that FKSUDDAPre exhibits significant advantages in the drug-disease association prediction task. The integration of XGBoost, Decision Tree, Random Forest, and HyperFast endows the model with a balance of accuracy, interpretability, and robustness. In multiple benchmarks, the average AUC of the ensemble model was significantly higher than that of any single base model. The introduction of the AMDKSU undersampling strategy and the F-test algorithm markedly improved model performance and enhanced the average discriminative power of the features. In comparative experiments with other methods, our model achieved an average AUC of 0.9725, with a performance improvement of approximately 3.88% compared to the best-performing baseline, further confirming the model’s superiority. In terms of validation of clinical significance, we selected Alzheimer’s disease (AD) and Parkinson’s disease (PD) as case studies and conducted a systematic literature review on the top 10 candidate drugs predicted by the model. The results showed that 8 candidate drugs for AD (80%) and 6 candidate drugs for PD (60%) were supported by PMID literature, further validating the model’s practical application potential.

Although the method proposed in this study has shown superior performance across multiple tasks, there is still room for improvement. First, to address the O(n²) computational complexity that may become a bottleneck in very large datasets, future work will explore the integration of Approximate Nearest Neighbor (ANN) search techniques to improve scalability. Second, while the F-test provides an efficient and interpretable mechanism for identifying key features, it primarily captures linear dependencies and may be limited in modeling nonlinear or interacting feature patterns. Future extensions will therefore consider complementary strategies—such as mutual-information–based criteria, ReliefF, or embedded feature selection methods derived from tree-based and neural models—to enhance the expressive capacity of the feature selection pipeline. In addition, we acknowledge that during dataset construction, drugs and diseases with fewer than ten known associations were excluded to ensure model stability and reduce noise. Although this strategy improves reliability, it may limit the model’s ability to generalize to cold-start drugs or rare diseases. Future studies will address this challenge through transfer learning and inductive graph-based approaches. Finally, we plan to extend our method to additional datasets and task scenarios, systematically evaluating its generalization ability and adaptability to promote real-world applicability.

## Supporting information

S1 Text Description of the online prediction tool. (DOCX)
