# Supplementary material for: FKSUDDAPre: A drug–disease association prediction framework based on F-TEST feature selection and AMDKSU resampling with interpretability analysis
Source: PLoS Comput Biol. 2026 Feb 5;22(2):e1013947. doi: 10.1371/journal.pcbi.1013947 (PMC12900433; doi:10.1371/journal.pcbi.1013947)
Supplement: S1 Text — (DOCX) [file pcbi.1013947.s001.docx]

To facilitate the online application and prediction capabilities of the proposed model, we developed a Python-based online prediction tool featuring a Graphical User Interface (GUI). The tool utilizes PyQt5 (v5.15.9) to implement the front-end user interface, and the executable (.exe) file of the predictor has been uploaded to GitHub for public download. The operational workflow of the predictor is as follows: users first load a locally stored pre-trained model in .pkl format via the "Select Model" button at the top of the interface, and subsequently import the target dataset in .csv format by clicking the "Select Data" button. The core prediction task is executed using the scikit-learn framework (v1.3.2), with prediction results returned and displayed on the user interface in real-time. The result list comprises four columns: Drug Index, Disease Index, Prediction Score, and Prediction Label (indicated as "negative" or "positive"). Furthermore, the tool integrates the pandas library (v2.1.1), enabling users to conveniently save the complete prediction results as a local .csv file.
